# Simple oral mucosal epithelial transplantation (SOMET) for ocular surface reconstruction in Stevens-Johnson Syndrome: A case report

**DOI:** 10.1016/j.ijscr.2023.108643

**Published:** 2023-08-09

**Authors:** Mariya B. Doctor, Raksheeth N. Rajagopal, Sayan Basu

**Affiliations:** a Shantilal Shanghvi Cornea Institute, L V Prasad Eye Institute, Kallam Anji Reddy Campus, Hyderabad, Telangana, India; b Prof. Brien Holden Centre for Eye Research (BHERC), L V Prasad Eye Institute, Hyderabad 500034, Telangana, India

**Keywords:** Stevens-Johnson Syndrome, Simple oral mucosal epithelial transplantation, Limbal stem cell deficiency, LSCD, Case report

## Abstract

**Introduction:**

We report the clinical outcome of a novel surgical technique called simple oral mucosal epithelial transplantation (SOMET) for the treatment of limbal stem cell deficiency (LSCD) in a patient with Stevens-Johnson Syndrome (SJS).

**Presentation of case:**

An eighteen-year-old girl was diagnosed as having chronic bilateral ocular sequelae of SJS. She initially underwent mucous membrane grafting (MMG) in both eyes for lid margin keratinization. Over the course of the next decade, the ocular surface cicatrization worsened in her left eye, leading to progressive symblepharon formation with total corneal conjunctivalization. She then underwent ocular surface reconstruction using bulbar MMG and SOMET. Following SOMET, the ocular surface epithelialized within 3 weeks and remained stable throughout the follow-up period. At one-year postoperatively, the visual acuity had improved from light perception to 20/250 unaided, and to 20/100 with scleral contact lens correction in the left eye.

**Discussion:**

Simple limbal epithelial transplantation (SLET) has been a boon for the treatment of unilateral LSCD. Allogeneic SLET and kerato-limbal allografts can be useful for patients with bilateral disease, however this exposes the patients to the risks of long-term systemic immunosuppression. SOMET combines the benefits of cultivated oral mucosal epithelial transplantation (COMET) and SLET, and is an autologous and single-staged surgical alternative for patients with bilateral LSCD.

**Conclusion:**

This case demonstrates that SOMET is a viable surgical option in cases with bilateral LSCD, eliminating the need for an allogeneic limbal graft, systemic immunosuppression, or laboratory cell culture.

## Highlights

• Simple oral mucosal epithelial transplantation (SOMET) is a novel option for bilateral limbal stem cell deficiency (LSCD).
• SOMET effectively restored the ocular surface and vision in a case of severe LSCD following Stevens-Johnson syndrome.
• SOMET is autologous and avoids the need for allogeneic limbal grafts, systemic immunosuppression, or laboratory cell culture.

## 1 Introduction

Stevens-Johnson Syndrome (SJS) is a rare, life-threatening, vesiculo-bullous disease of the skin and mucosa, occurring due to an immune-mediated hypersensitivity reaction to a systemic drug or infection [1]. Ocular involvement is the most common chronic sequelae of SJS, which impairs the quality of life of affected individuals [1,2]. Keratopathy and limbal stem cell deficiency (LSCD) ensue due to constant friction from lid margin keratinization, long-standing ocular surface inflammation, conjunctival cicatrization, and aqueous deficient dry eye disease, leading to sight-threatening complications [2]. Treatment of lid margin keratinization involves lid margin mucous membrane grafting (MMG) or the use of prosthetic replacement of ocular surface ecosystem (PROSE) contact lenses [[3], [4], [5]]. The fornix and the ocular surface can be reconstructed using amniotic membrane transplantation (AMT), ocular surface MMG, or cultivated oral mucosal epithelial transplantation (COMET) [6,7]. Bilateral LSCD in these patients can be treated with allogeneic limbal stem cell transplantation or COMET [8]. Being an autologous procedure, COMET carries no risk of immunological rejection, improves visual acuity, ocular surface severity scoring, and helps treat LSCD [9]. However, it requires a sophisticated and expensive clinical-grade laboratory setup. Allogeneic limbal stem cell transplantation, on the other hand, requires long-term systemic immunosuppression. In this case report, we applied the concept of simple limbal epithelial transplantation (SLET) and combined it with the benefits of COMET, by replacing the limbal tissue with autologous oral mucosa, using a surgical technique termed simple oral mucosal epithelial transplantation (SOMET). This case report is as per the SCARE-2020 criteria [10].

## 2 Presentation of case

**Fig. 1:**
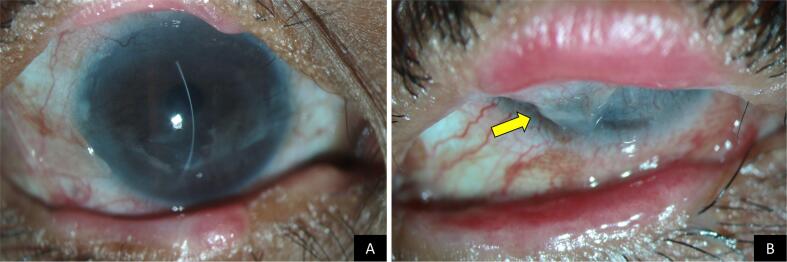
Slit lamp photographs of both eyes around ten years after of undergoing eyelid mucous membrane grafting. The right eye (A), which had also undergone symblepharon release with amniotic membrane transplantation and cataract surgery two years prior shows: thickened lid margins, integrated eyelid mucous membrane grafts in both upper and lower eyelid, with nebular grade corneal scarring. The left eye (B) shows: thickened lid margins with mucous membrane grafts in place, an extensive symblepharon covering almost the entire cornea (yellow arrow), limiting eversion of the upper lid and precluding the view of the cornea. (For interpretation of the references to colour in this figure legend, the reader is referred to the web version of this article.)

An eighteen-year-old girl presented to us in 2012 with complaints of photophobia, redness, and discharge in both eyes (OU) for the past three years, after an episode of multiple skin eruptions and rashes over the body, which occurred as a consequence of consuming systemic medication for fever. At presentation, she had a best corrected visual acuity (BCVA) of 20/40p in OU. There were blocked meibomian orifices, thickened and keratinized lid margins, conjunctival cicatrization, and corneal scarring with vascularization noted in OU. She was diagnosed as having ocular sequelae of SJS and was started on topical steroids, and frequent application of tear substitutes. She later underwent lid margin MMG in OU. The right eye (OD) later underwent symblepharon release, amniotic membrane grafting and cataract surgery. While OD was relatively stable (Fig. 1A), in the left eye (OS) over the period of the next ten years, she developed a progressive symblepharon covering 80–90 % of the cornea (Fig. 1B) causing her visual acuity to drop from 20/40 to light perception. At this stage we intervened surgically in OS and performed symblepharon release with ocular surface reconstruction using bulbar MMG and SOMET.

**Fig. 2:**
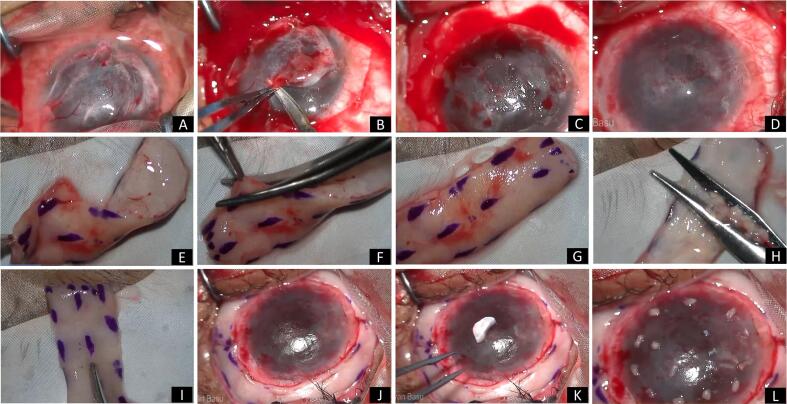
Surgical technique of simple oral mucosal epithelial transplantation or SOMET (A to L). A: The symblepharon was excised (A) and the self-retaining speculum was placed to expose the cornea and ocular surface. A 360-degree peritomy was performed (B) and the fibro-vascular pannus was dissected from the underlying corneal stroma (C). An amniotic membrane graft was placed over the ocular surface using fibrin glue, along with anchoring, perilimbal, continuous sutures with 10-0 nylon (D). The oral mucous membrane graft was harvested from the lower lip (E), and a small triangular shaped tissue was excised (F, G) and kept aside, for later use as SOMET. The graft was thinned to the extent possible by excising the underlying submucosal tissue and fat (H). The tissue was then dissected into two equal parts (I). The grafts were sutured over the bulbar bare sclera, circumferentially around the limbus (J), using 7-0 vicryl sutures (K). The triangular tissue was then thinned (K) and divided into 10–15 small pieces, and placed over the cornea, using fibrin glue (L).

Surgical procedure (Fig. 2): under general anesthesia, after painting and draping the eye, the symblepharon was released from the cornea and a 360-degree peritomy was performed (Fig. 2A, B). A combination of sharp and blunt dissection was carried out to separate the fibrovascular pannus from the underlying corneal stroma (Fig. 2C). An amniotic membrane graft (AMG) was attached over the ocular surface with fibrin glue and perilimbal 10-0 nylon continuous sutures (Fig. 2D). The oral mucosa over the lower lip was cleaned with betadine 5 % solution. The submucosal tissue was infiltrated with 5 mL of lignocaine hydrochloride 2 % and adrenaline (1:200,000) solution. The MMG was marked with tissue markers and borders incised with number 11 blade. It was then carefully excised and placed over the surgical drape (Fig. 2E). A small triangular piece of the MMG tissue (approximately 3 mm in size) was excised and kept aside for SOMET later (Fig. 2F). The submucosal tissue was debulked, and the graft was thinned to the extent possible (Fig. 2G, H). It was divided into 2 horizontally equal parts for surface reconstruction (Fig. 2I). The grafts were attached circumferentially over the sclera around the limbus using 7-0 vicryl sutures (Fig. 2J–K). The remaining triangular tissue of MMG (Fig. 2K) was thinned and divided into 10–15 tiny pieces, which were placed over the paracentral cornea (similar to SLET), using fibrin glue, over which a soft bandage contact lens (BCL) was placed (Fig. 2L).

**Fig. 3:**
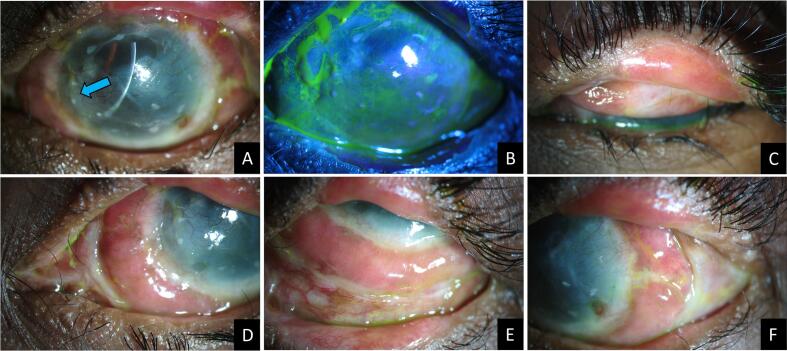
Slit lamp photographs of the left eye three weeks after simple oral mucosal epithelial transplantation or SOMET (A to F). The tiny pieces of mucosal grafts (A, blue arrow) can be appreciated in the paracentral cornea. Fluorescein-stained image in cobalt blue filter (B) shows a completely epithelized corneal surface. Everted image of the left upper eyelid (C) reveals a deep upper fornix. Images in different gazes (D to F) showing 360-degree bulbar mucous membrane grafts, which are well taken up. (For interpretation of the references to colour in this figure legend, the reader is referred to the web version of this article.)

**Fig. 4:**
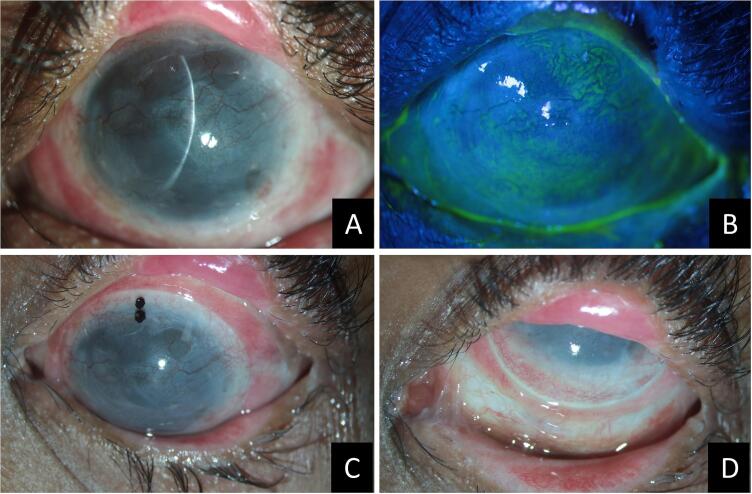
Slit lamp photos of the left eye at the one-year follow-up visit after simple oral mucosal epithelial transplantation or SOMET (A to D). The corneal surface is stable (A), completely epithelized (B) with completely integrated SOMET grafts and macular to nebular grade superficial corneal scarring and vascularization. Images after wearing PROSE contact lenses in the left eye (C, D) which show acceptable fitting of the lenses over the reconstructed corneal and bulbar surface.

Post-operatively, she was followed up at 1 day, 1 week, 3 weeks, 2 months, 6 months, and 1 year. She was started on low-dose oral steroids (20 mg/day) for a week, which were tapered to 5 mg/day for a month and stopped thereafter; topical tear substitutes, and ointment of steroid and antibiotic combination applied thrice a day for 1 week and later shifted to only nightly application. The cornea had completely epithelized by 3 weeks (Fig. 3A, B) and the BCL was removed. The fornices had been restored (Fig. 3C) and the ocular surface (bulbar) MMG had also integrated well (Fig. 3D–F). We encountered no epithelial instability, or any other complication during the entire post-operative period. At the final 1-year follow-up visit, the ocular surface was stable, the cornea was completely epithelized, the SOMET grafts had completely integrated, and there was residual macular to nebular grade superficial stromal scarring with vascularization (Fig. 4A, B). The uncorrected visual acuity was 20/200 and 20/250 in OD and OS, respectively. She was improving to 20/100 in OS with PROSE contact lenses (Fig. 4C, D). The Schirmer's test value had also increased from 0 mm at 5 min pre-operatively to 6 mm post-operatively.

## 3 Discussion

Following SJS, patients usually present to an ophthalmologist after developing ocular symptoms such as foreign body sensation, dry eyes, or decrease in visual acuity, months to years after the acute episode [11]. The chronic stage may lead to sight-threatening complications due to lid margin keratinization, dry eye disease, and chronic ocular surface inflammation [[2], [3], [4], [5], [6],^11^]. Since LSCD in SJS is often bilateral, surgical options include allogeneic limbal stem cell transplantation in the form of allo-SLET, or keratolimbal allografting (KLAL), or autologous COMET. Outcomes of limbal allografting in cases of SJS have not shown to be satisfactory due to high chances of failure and the need for long-term immunosuppression which have other potential side effects [11,12]. COMET has a success rate of about 70 % in cases of bilateral LSCD. It helps in rapid surface epithelization, reduces corneal opacification, and improves visual acuity [9,13,14]. Studies have shown that the cultured cells resemble the corneal epithelial cell having three to five cell layers, with small basal cells, flattened middle cells, and polygonal superficial cells. They express markers of corneal epithelial cell differentiation (cytokeratin 3 and connexin 43), and marker of progenitor stem cells (p63), similar to corneal epithelial cells [[13], [14], [15]].

COMET has the advantage of being an autologous procedure, and not requiring any systemic immunosuppression; while the disadvantages include it being a two-staged procedure, and the requirement of an expensive clinical-grade laboratory set-up for cell cultivation [8]. Based on the encouraging outcomes of COMET, and combining them with the principle of SLET (where autologous limbal tissue from a healthy eye is divided into multiple small pieces and placed over the AMG in the paracentral cornea for these cells to proliferate and eventually epithelize and cover the entire corneal surface) [16,17] we decided to perform SOMET, a synthesis of both these techniques. We placed tiny pieces of the MMG, obtained from the lip mucosa, circumferentially at the paracentral cornea with the help of fibrin glue over an underlying AMG. These transplants were seen as discrete tissue initially but later integrated and led to complete epithelization of the corneal surface by 3 weeks. Post-operatively, a well epithelized surface, improvement in visual acuity and Schirmer's value were noted at 1-year follow-up in our patient.

There are few reports of the oral mucosa being used as a tissue graft, without ex-vivo cultivation as in COMET, for the treatment of LSCD [18,19]. In a retrospective study done by Liu et al., an oral mucosal graft was used as a surrogate limbus in 7 eyes with severe LSCD (1 eye having LSCD due to SJS). They too simultaneously treated the symblepharon along with LSCD. Their results showed that all patients had improvement in symptoms and corneal scarring. They also demonstrated that initially, the peripheral corneal vascularization increased, which then decreased significantly after 3–6 months [18]. The first report of performing SOMET was in a rabbit model of LSCD by Inamochi et al. [19]. In this study a 3 × 4 mm piece or oral mucosal tissue was excised and divided into small pieces, treated with dispase II, and placed over the denuded cornea, without AMT or fibrin glue. They demonstrated that the epithelium expanded to form islands around the grafts 1 week after surgery and the corneal defect was completely epithelialized at 2 weeks, similar to the results of SLET. They went on to describe that the single surface layer of epithelial cells was CK3-positive, and basal cells were p63 positive similar to corneal epithelial cells [19]. Hence, they concluded that the direct placement of autologous oral mucosal tissue, without ex-vivo cell culture, could be a good alternative to limbal allografting in bilateral LSCD. This report is the first clinical validation of the SOMET technique proposed by Inamochi et al. The modifications from the animal study were that there was no prior treatment of the mucosal tissue with dispase, we used AMG on the cornea, and used fibrin glue to secure the transplants, analogous to SLET for the treatment of LSCD. Here the AMG would act as a scaffold for the mucosal epithelial cells to proliferate rapidly. We also performed simultaneous bulbar surface reconstruction with MMG to address the severe symblepharon, analogous to a conjunctival autograft or MMG being performed along with SLET to address the conjunctival deficiency [17]. This approach was not only effective in anatomically restoring the ocular surface but also resulted in significant functional improvement in unaided and contact lens-corrected visual acuity.

## 4 Conclusion

This study highlights the potential of SOMET as a surgical alternative to allogeneic limbal transplantation and COMET for the treatment of bilateral LSCD. It combines the strengths of SLET and COMET, while avoiding the need for long-term systemic immunosuppression or laboratory cell cultivation. However, a prospective study with a larger sample size is required to validate the long-term clinical outcomes of SOMET in different cases of bilateral LSCD.

## Consent

Written informed consent was obtained from the patient for publication of this case report and accompanying images. A copy of the written consent is available for review by the Editor-in-Chief of this journal on request.

## Patient perspective

“I was blind and disfigured in my left eye. The surgery helped me in seeing better, but what I was most impressed with was the improvement in the cosmetic appearance of the eye. This has helped improve my self-confidence and self-esteem.”

## Declaration of competing interest

The authors have no conflict of interest.
